# Supplementary material for: TGFβ suppresses CD8+ T cell expression of CXCR3 and tumor trafficking
Source: Nat Commun. 2020 Apr 9;11:1749. doi: 10.1038/s41467-020-15404-8 (PMC7145847; doi:10.1038/s41467-020-15404-8)
Supplement: Supplementary file 1 — Supplementary Information [file 41467_2020_15404_MOESM1_ESM.pdf]

TGFβ suppresses CD8$^+$ T cell expression of CXCR3 and tumor trafficking

Gunderson AJ, et al.

Supplementary Information

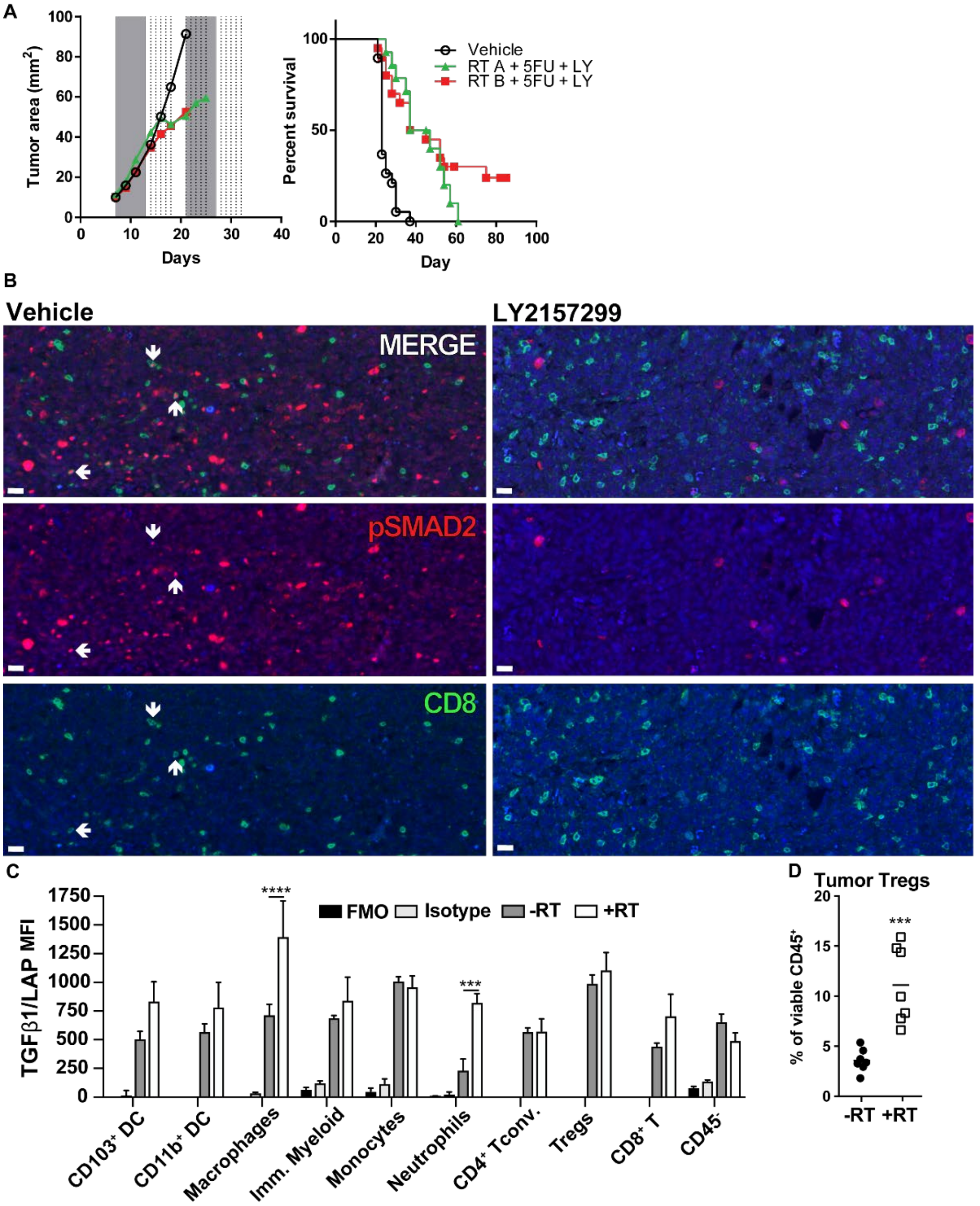

Supplementary Figure 1: TGFβ1 produced by numerous cell types can be blocked using LY2157299 to improve treatment efficacy. A) Tumor growth (i) and survival (ii) using 5Gy x 5 (RT A, green triangles) and 2Gy x 15 (RT B, red squares) with 5-fluorouracil (5FU) and LY2157299 (LY). Days of radiation delivery dashed, grey bars represent days of LY administration consistent with Fig. 1A. n=22-23/group. Combined analysis of 3 independent experiments. For (i), p-values are a comparison between control and RT A + 5FU + LY, as well as control and RT B + 5FU + LY, using an unpaired, two-tailed t-test. *p=0.0077 (green triangle vs open circle), **p=0.018 (green triangle vs open circle) and p=0.019 (red square vs open circle), ***p=0.00026 (green triangle vs open circle) and p=0.0011 (red square vs open circle). For (ii), p-values were derived using long-rank test. vehicle vs RT A+5FU+LY<0.0001(****),vehicle vs RT B+5FU+LY<0.0001(****),RT A+5FU+LY vs RT B+5FU+LY p=0.433(NS). B) Representative phospho-SMAD2 (pSmad2, red) and CD8α$^+$ (green) co-immunofluorescent images from tumors harvested from mice at day 14 prior to cytotoxic therapy. Arrows identify pSMAD2$^+$CD8$^+$ cells in mice receiving the vehicle control. Shown is a representative image reflective of three independent experiments. Scale bar = 20 micrometers. C) Intracellular TGFβ1/LAP expression was determined by FACS analysis of MC38 tumors from control unirradiated mice (-RT) and radiated (RT) mice, 10Gy x 2 on days 14 and 15, with tumor harvest on day 21. Cells gated on singlet, viable CD45$^+$ cells and the indicated populations as follows: CD103$^+$ DC=CD11b$^-$CD11b$^+$MHCII$^+$CD103$^+$, CD11b$^+$ DC=CD11b$^+$MHCII$^+$CD11c$^+$F4/80$^-$, Macrophages=CD11b$^+$F4/80$^+$MHCII$^+$, Immature Myeloid=CD11b$^+$MHCII$^-$Ly6C$^-$Ly6G$^-$,

Monocytes=$CD11b^+MHCII^-Ly6C^+Ly6G^-$, Neutrophils=$CD11b^+MHCII^-Ly6C^+Ly6G^+$, $CD4^+$ T conventional cells=$CD3^+CD4^+CD8^-Foxp3^-$, Tregs=$CD3^+CD4^+CD8^-Foxp3^+$, $CD8^+$ T cells=$CD3^+CD4^-CD8^+$; average TGFβ1/LAP MFI is calculated in each subset. *p=0.0277, ***p=0.0002. N=5/group. Measure of center = mean. Shown is one experiment reflective of two independent experiments. P-value was determined using 1-way ANOVA with multiple comparisons. D) The frequency of $CD3^+CD4^+Foxp3^+$ Tregs in MC38 tumors was determined by FACS analysis treated as in C. ***p=0.00031. N=7/group. Measure of center = mean. Shown is one experiment reflective of two independent experiments. P-value was determined using student's unpaired, two-tailed t-test.

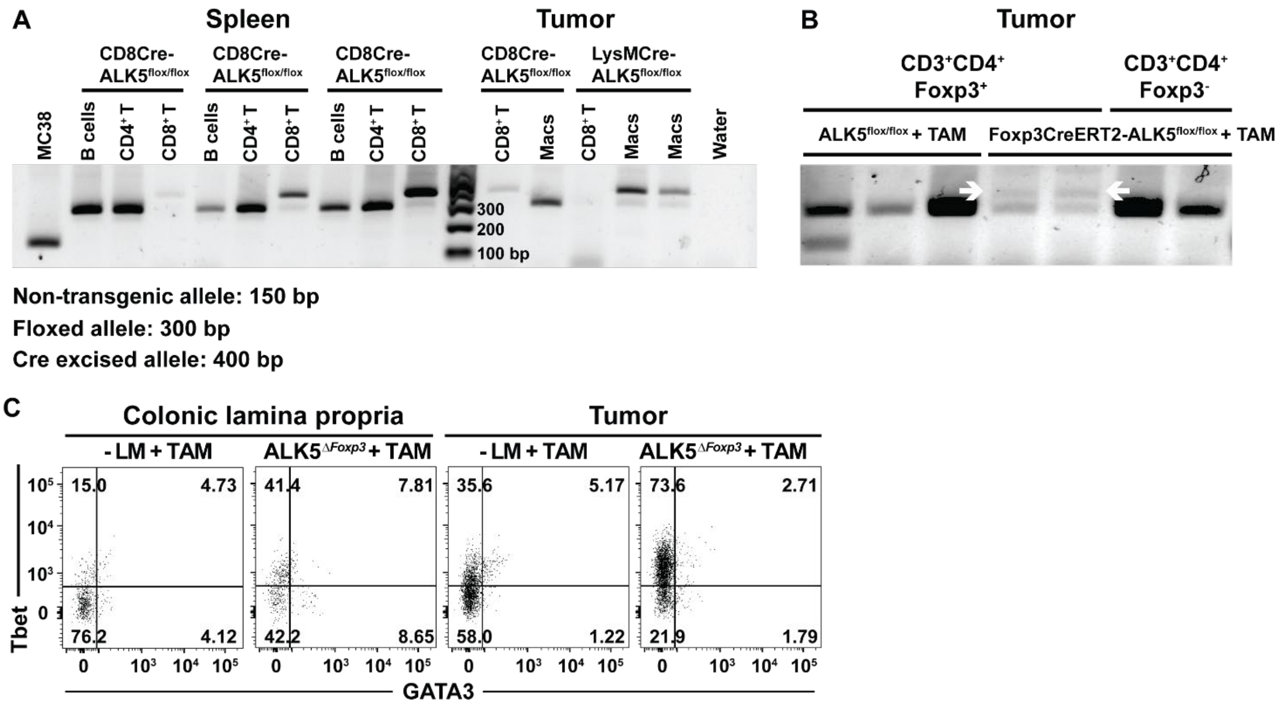

Supplementary Figure 2: Cell type-specific ALK5 gene excision following conditional Cre expression and resultant alteration of Tbet expression in Tregs. A) PCR analysis of ALK5 gene regions following cell sorting for CD19$^+$ B cells, CD4$^+$ T cells, CD8$^+$ T cells and macrophages in spleens and MC38 tumors in CD8Cre-ALK5$^{flox/flox}$ (n=3) and Lyz2Cre-ALK5$^{flox/flox}$ mice (n=2). DNA from MC38 tumor cells and water blank are negative controls. B) ALK5 PCR on DNA from FACS purified Foxp3$^+$ Tregs and Foxp3$^-$ conventional CD4$^+$ T cells was harvested from 21-day MC38 tumors in ALK5$^{flox/flox}$ single transgenic (n=3) or Foxp3-eGFPCreERT2/ALK5$^{flox/flox}$ mice (n=2). Tamoxifen was administered for 5 consecutive days prior to implant to induce Cre recombination. White arrowheads identify Cre excised alleles. C) FACS analysis on single cell suspensions prepared from colons and tumors of single transgenic negative littermates or double transgenic ALK5$^{\Delta Foxp3}$ mice when tumors reach 144 mm2. Shown is the expression of the transcription factors Tbet and GATA3 in Foxp3$^+$ Tregs in the indicated tissue. All data shown is 1 representative experiment reflective of 2 total.

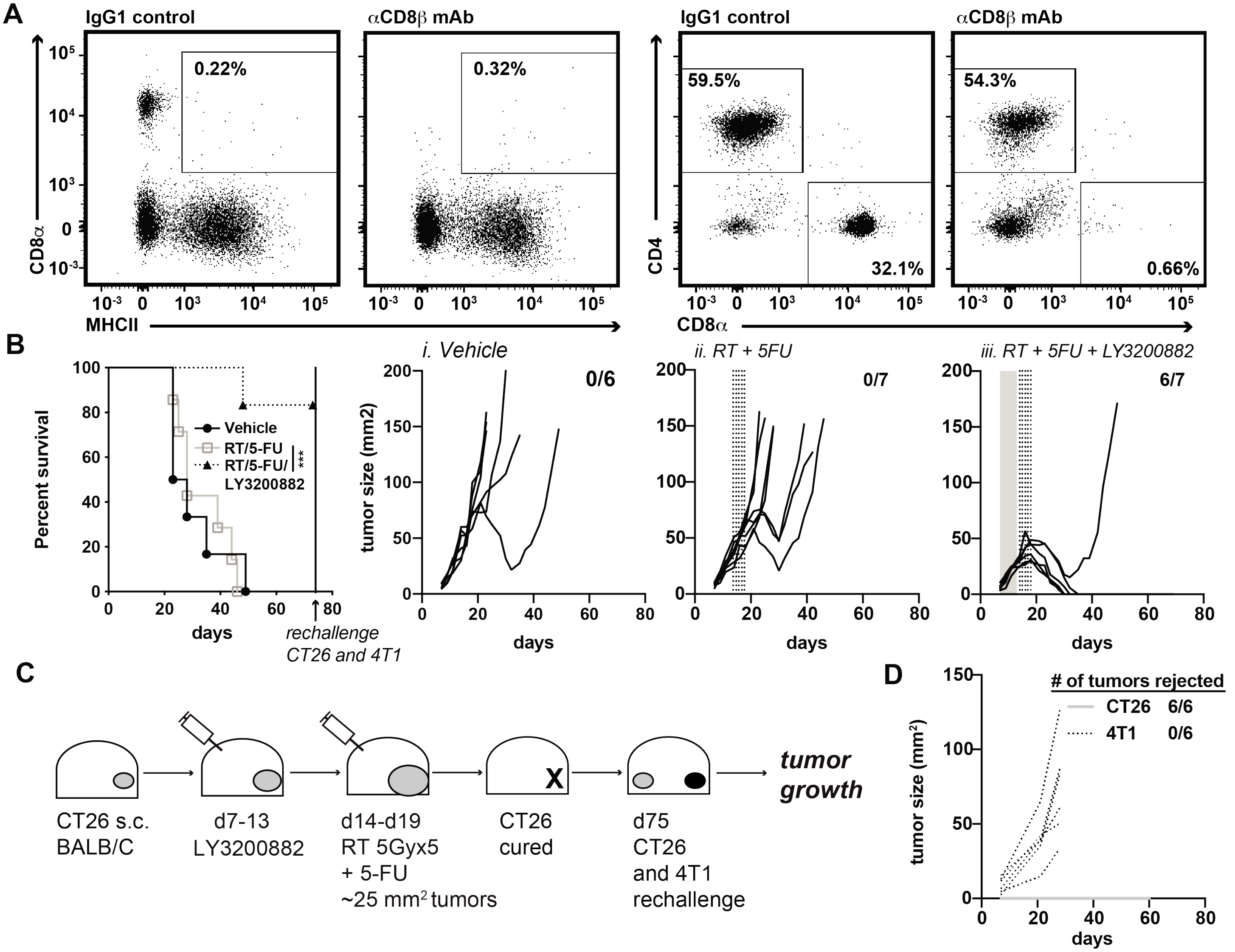

Supplementary Figure 3: Development of adaptive, tumor-specific immune memory following tumor eradication with TGFβ inhibition and chemoradiation. A) Representative FACS plots from spleens of MC38 tumor-bearing mice harvested 14 days following injection with depleting anti-CD8β or IgG1 isotype control. Shown on the left is CD8α vs. MHCII expression gated on viable $CD45^+$ cells and shown on the right is CD4 vs. CD8α expression gated on viable $CD45^+CD3^+$ cells. B) Survival and individual tumor growth curves in CT26 tumor bearing mice treated with i) vehicle control, ii) chemoradiation (RT/5-FU) on days 14-19, or iii) chemoradiation + LY320082 from d7-13 and d21-27 (RT/5-FU/LY320082). The number of mice cured over the total number of mice in each group is indicated in the top right corner. One representative experiment is shown reflective of two independent experiments. P-value derived using log-rank test. ***p=0.0009. C) Treatment outline for *B* and *D.* Mice whose tumors were eradicated were rechallenged with CT26 and 4T1 tumor cells. CT26 tumor cells were implanted on the opposite flank from the original cured tumor. D) 4T1 and CT26 tumor establishment and growth were followed for up to 60 days post re-challenge. The number of tumors rejected per total mice re-challenged in each tumor type is indicated in the top right portion of the graph.

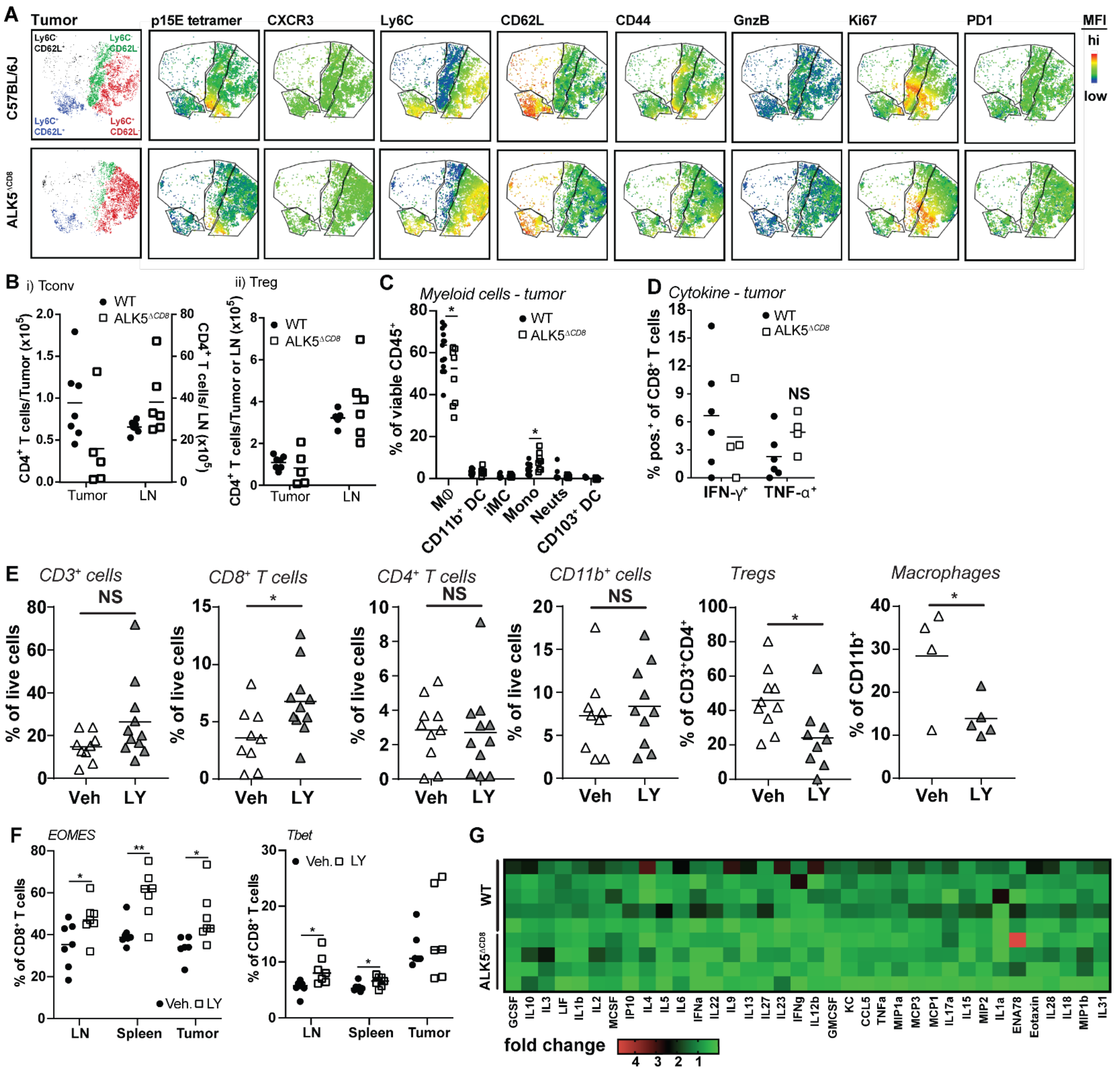

Supplementary Figure 4: Tumor immune cell infiltrate with ALK5 inhibition is similar to that observed in ALK5$^{\Delta CD8}$ mice. A) Representative tSNE plots of CD8$^+$ T cells generated by FACS analysis of MC38 tumors from C57BL/6 or ALK5$^{\Delta CD8}$ mice. Gates are populations of Ly6C$^{+/-}$CD62L$^{+/-}$ overlaid in color on far left. Heat maps represent the minimum to maximum MFI value for each marker. B-D) FACS analysis of day 14 MC38 tumor digests from WT (black dots) and ALK5$^{\Delta CD8}$ mice (open square). B) Absolute numbers of i) CD4$^+$Foxp3$^-$ T conventional cells and ii) CD4$^+$Foxp3$^+$ T regulatory cells in the tumor (left) and draining lymph node (right). n=5-7 mice/group, one experiment representative of 2 independent experiments, measurement of center=mean. C) FACS analysis of myeloid cell subsets in the tumor of WT (n=12) and ALK5$^{\Delta CD8}$ (n=8) mice. Measurement of center = mean. *p=0.0463 (macrophages), *p=0.0314 (monocytes). Combined analysis of 2 independent experiments. D) IFN-γ$^+$ or TNF-α$^+$ CD8$^+$ T cells in MC38 tumors in WT (n=6) and ALK5$^{\Delta CD8}$ (n=4) mice determined by FACS intracellular cytokine staining analysis. Measurement of center = mean. Representative experiment reflective of two independent experiments. E) FACS analysis of day 14 tumors from vehicle and LY treated mice, 24 hours after last LY dose. Frequency of total CD3$^+$ T cells, CD8$^+$ T cells (p=0.0225), CD4$^+$ T cells, CD11b$^+$ cells of viable cells, and Tregs of CD4$^+$ T cells (p=0.0181), macrophages of CD11b$^+$ myeloid cells (p=0.0379). Displayed as mean+/-SD. N=10 mice/group, except macrophage n=4-5 mice/group. Combined results of 2

independent experiments. F) FACS analysis of lymph nodes(LN), spleens, and tumors from CT26 tumor-bearing mice treated with vehicle (n=7) or LY3200882 (n=7) for 7 days. Percent EOMES (D) or Tbet (E) positive of $CD8^+$ T cells, measure of center = mean. *p=0.0418 (EOMES, LN), **p=0.00249 (EOMES, spleen), *p=0.0238 (EOMES, tumor); *p=0.0153 (Tbet, LN), *p=0.0407 (Tbet, spleen). One representative experiment of 2 independent experiments. G) Cytokine protein levels tumor lysates derived from day 14 MC38 tumors in C57BL/6 (n=5) and $ALK5^{\Delta CD8}$ (n=4) mice, representative experiment reflective of two independent experiments. Protein amount is expressed as a fold change in pg cytokine/total mg protein normalized to WT tumors and displayed as a heat map for individual animals. Markers used for FACS analysis are the same as described in Supp. Fig 1B. For all panels, p-values were obtained using unpaired, two-sided t-test.

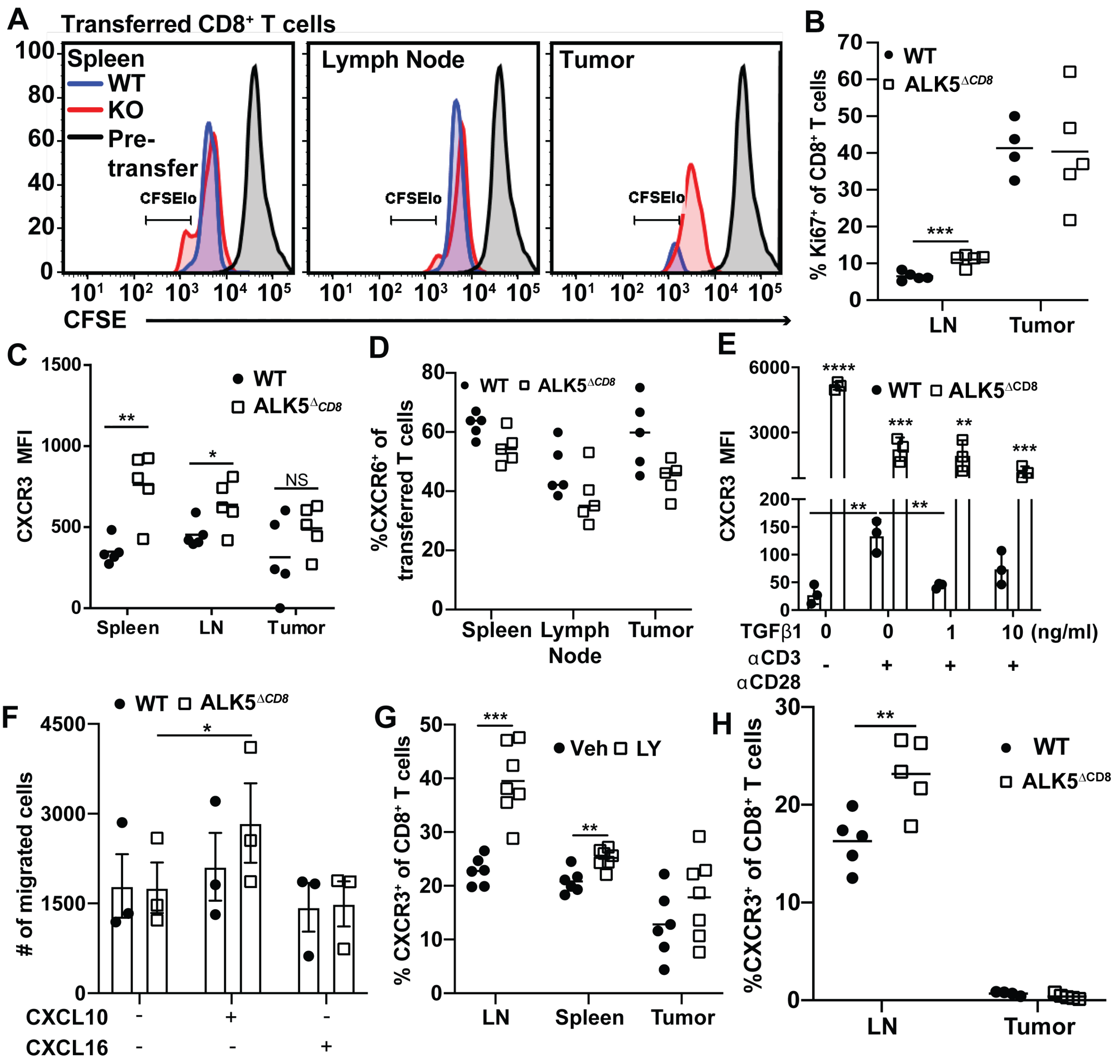

Supplementary Figure 5: CD8+ T cell proliferation is suppressed by the tumor microenvironment, but TGFβ inhibition can increase tumor trafficking. A) Representative histograms of CFSE fluorescence in transferred CD8+ T cells of WT (blue) and KO (red) cells overlaid and compared to the CSFE fluorescence of the mixed population prior to pre-transfer (grey). B) Ki67+CD8+ T cells was determined by FACS analysis of lymph nodes and MC38 tumors of WT (n=5) and ALK5$^{\Delta CD8}$ (n=5) mice. Representative experiment reflective of two independent experiments. ***p=0.000858. C) CXCR3 MFI of transferred cells. Measure of center = mean. N=5 recipients/group, representative experiment reflective of two independent experiments. **p=0.00289, *p=0.041. D) CXCR6+CD8+ T cells were determined by FACS analysis of spleen, lymph nodes and MC38 tumors from adoptively transferred WT and ALK5$^{\Delta CD8}$ CD8+ T cells from *A*. Measure of center = mean. N=5 recipients/group, representative experiment reflective of two independent experiments. E) CXCR3 MFI of naïve splenocyte-derived CD8+ T cells from C57BL/6 or ALK5$^{\Delta CD8}$ mice stimulated with plate-bound αCD3/αCD28 +/- TGFβ1 (1 or 10 ng/ml) for 68 hours; n=3 biologic replicates/group, displayed as mean+/-SD. For WT vs ALK5$^{\Delta CD8}$: ****p= 0.0000010 (unstimulated,), ***p=0.00247 (0ng/mL), **p=0.010 (1ng/mL), ***p=0.00186 (10ng/mL). For WT vs WT: **p=0.0055 (unstim vs 0ng/mL), **p=0.0060 (0ng/mL vs 10ng/mL). F) In vitro

migration assay of WT and ALK5$^{\Delta CD8}$ $CD8^+$ T cells in response to CXCL10 and CXCL16 treatment in the bottom chamber. n=3 biologic replicates/group, displayed as mean+/-SD. Representative experiment reflective of two independent experiments. *p=0.04735.G) FACS analysis of $CXCR3^+CD8^+$ T cells from lymph nodes, spleens, and tumors from CT26 tumor bearing mice treated with vehicle control (n=6) or LY3200882 (n=7) for 7 days, displayed as mean+/-SD. Representative experiment reflective of two independent experiments. **p=0.00197, ***p=0.000133. H) Endogenous CXCR3+ of CD8+ T cells from MC38 tumor-bearing WT (n=5) and ALK5$^{\Delta CD8}$ (n=5) mice at day 21, measure of center=mean. Representative experiment reflective of two independent experiments **p=0.0011. One-way ANOVA with multiple comparisons was used to determine significance in panels E and F, all other panels used unpaired, two-tailed t-test.

Supplementary Table 1. Antibodies for flow cytometry

Antigen	Fluorescent conjugate	Dilution	Vendor
CD4	BV605	1:400	BD Biosciences
CD8α	APC-Cy7	1:400	BD Biosciences
CD3e	PerCP-EF710	1:200	eBioscience
CD45	BV510	1:400	BD Biosciences
CD11b	PE-Cy7, BV605	1:5000, 1:1000	BD Biosciences
MHCII (IA-IE)	EF450	1:1000	eBioscience
CD11c	APC-Cy7, PE-Cy7	1:400	BD Biosciences
IFNγ	APC	1:400	BD Biosciences
Ki67	APC	1:400	eBioscience
GnzB	FITC	1:200	BD Bioscience
P15E tetramer	PE	1:2000	NIH tetramer core
TNFα	PE-Cy7	1:400	BD Bioscience
CD103	PE, APC	1:400	BD Biosciences
F4/80	APC	1:400	BD Biosciences
CD44	BV711	1:800	BD Biosciences
CD62L	PE-Cy7	1:800	eBioscience
Ly6C	PerCP-Cy5.5	1:400	eBioscience
Ly6G	FITC	1:500	BD Biosciences
PD-1	BV786	1:200	BD Biosciences
Tbet	BV650	1:200	BD Biosciences
EOMES	PE	1:200	eBioscience
CD80	PE-CF594	1:400	eBioscience
CD40	FITC	1:200	BD Biosciences
Foxp3	EF450	1:200	eBioscience
GATA3	EF660	1:200	eBioscience
CXCR3	BV421	1:200	BD Biosciences
CXCR6	BV711	1:200	Biolegend
KLRG1	PE-DAZZLE 594	1:200	Biolegend
CD4 (human)	PerCP-Cy5.5	1:50	BD Biosciences
CD3 (human)	AF700	1:75	BD Biosciences
CXCR3 (human)	PE-CF594	1:150	BD Biosciences
CD45 (human)	FITC	1:100	BD Biosciences
