# Supplementary material for: TGFβ suppresses CD8+ T cell expression of CXCR3 and tumor trafficking
Source: Nat Commun. 2020 Apr 9;11:1749. doi: 10.1038/s41467-020-15404-8 (PMC7145847; doi:10.1038/s41467-020-15404-8)
Supplement: Supplementary file 3 — Reporting Summary [file 41467_2020_15404_MOESM3_ESM.pdf]

nature research

Corresponding author(s): Kristina Young

Last updated by author(s): 02/17/2020

Reporting Summary

Nature Research wishes to improve the reproducibility of the work that we publish. This form provides structure for consistency and transparency in reporting. For further information on Nature Research policies, see Authors & Referees and the Editorial Policy Checklist .

Statistics

For all statistical analyses, confirm that the following items are present in the figure legend, table legend, main text, or Methods section.

n/a	Confirmed	
[ ]	[x]	The exact sample size (*n*) for each experimental group/condition, given as a discrete number and unit of measurement
[ ]	[x]	A statement on whether measurements were taken from distinct samples or whether the same sample was measured repeatedly
[ ]	[x]	The statistical test(s) used AND whether they are one- or two-sided<br>*Only common tests should be described solely by name; describe more complex techniques in the Methods section.*
[ ]	[x]	A description of all covariates tested
[ ]	[x]	A description of any assumptions or corrections, such as tests of normality and adjustment for multiple comparisons
[ ]	[x]	A full description of the statistical parameters including central tendency (e.g. means) or other basic estimates (e.g. regression coefficient) AND variation (e.g. standard deviation) or associated estimates of uncertainty (e.g. confidence intervals)
[x]	[ ]	For null hypothesis testing, the test statistic (e.g. *F*, *t*, *r*) with confidence intervals, effect sizes, degrees of freedom and *P* value noted<br>*Give P values as exact values whenever suitable.*
[x]	[ ]	For Bayesian analysis, information on the choice of priors and Markov chain Monte Carlo settings
[x]	[ ]	For hierarchical and complex designs, identification of the appropriate level for tests and full reporting of outcomes
[ ]	[x]	Estimates of effect sizes (e.g. Cohen's *d*, Pearson's *r*), indicating how they were calculated

Our web collection on statistics for biologists contains articles on many of the points above.

Software and code

Policy information about availability of computer code

Data collection	Flow cytometry data was analyzed using commercially available FlowJo software. Data analysis was performed using Graphpad Prism 7.0 software.
Data analysis	Graphpad Prism 7.0 software was used to construct all graphs and calculate statistical significance. FlowJo software was used for FACS analysis and to generate tSNE plots.

For manuscripts utilizing custom algorithms or software that are central to the research but not yet described in published literature, software must be made available to editors/reviewers. We strongly encourage code deposition in a community repository (e.g. GitHub). See the Nature Research guidelines for submitting code & software for further information.

Data

Policy information about availability of data

All manuscripts must include a data availability statement. This statement should provide the following information, where applicable:

- Accession codes, unique identifiers, or web links for publicly available datasets
- A list of figures that have associated raw data
- A description of any restrictions on data availability

All data published is representative of experiments and replicates. RNAseq data was mined from TCGA – colorectal PanCancer Atlas data set on the cBioPortal for cancer genomics (cbioportal.org). SMAD2 and CXCR3 normalized mRNA expression levels were compared by linear regression analysis. Animal models, reagents, and software used are publicly available. There are no restrictions on our data.

Field-specific reporting

Please select the one below that is the best fit for your research. If you are not sure, read the appropriate sections before making your selection.

☒ Life sciences ☐ Behavioural & social sciences ☐ Ecological, evolutionary & environmental sciences

For a reference copy of the document with all sections, see nature.com/documents/nr-reporting-summary-flat.pdf

Life sciences study design

All studies must disclose on these points even when the disclosure is negative.

Sample size	The efficacy of therapy on time-to-failure will be determined by Kaplan-Meier survival curves compared using a log-rank test with significance of $p<0.05$. 8 mice/group provides 80% power to detect a difference in survival at four weeks post treatment of 50% vs. 84%, and difference in median survival of 1 week.
Data exclusions	Data was excluded from qPCR if >30 cycles were performed without amplification signal, or if >3 standard deviations from the mean with 3 or more replicates.
Replication	Every experiment was performed a minimum of two times. If results different between experiments, a third replicate was performed.
Randomization	Mice were randomized into groups prior to treatment to limit variation between groups.
Blinding	Tumor size measurements were performed by a blinded member of the research team.

Reporting for specific materials, systems and methods

We require information from authors about some types of materials, experimental systems and methods used in many studies. Here, indicate whether each material, system or method listed is relevant to your study. If you are not sure if a list item applies to your research, read the appropriate section before selecting a response.

Materials & experimental systems

n/a	Involved in the study	
☐	☒	Antibodies
☐	☒	Eukaryotic cell lines
☒	☐	Palaeontology
☐	☒	Animals and other organisms
☐	☒	Human research participants
☐	☒	Clinical data

Methods

n/a	Involved in the study	
☒	☐	ChIP-seq
☐	☒	Flow cytometry
☒	☐	MRI-based neuroimaging

Antibodies

Antibodies used

Antigen Fluorescent conjugate Dilution Vendor
CD4 BV605 1:400 BD Biosciences
CD8α APC-Cy7 1:400 BD Biosciences
CD3e PerCP-EF710 1:200 eBioscience
CD45 BV510 1:400 BD Biosciences
CD11b PE-Cy7, BV605 1:5000, 1:1000 BD Biosciences
MHCII (IA-IE) EF450 1:1000 eBioscience
CD11c APC-Cy7, PE-Cy7 1:400 BD Biosciences
IFNγ APC 1:400 BD Biosciences
Ki67 APC 1:400 eBioscience
GnzB FITC 1:200 BD Bioscience
P15E tetramer PE 1:2000 NIH tetramer core
TNFα PE-Cy7 1:400 BD Bioscience
CD103 PE, APC 1:400 BD Biosciences
F4/80 APC 1:400 BD Biosciences
CD44 BV711 1:800 BD Biosciences
CD62L PE-Cy7 1:800 eBioscience
Ly6C PerCP-Cy5.5 1:400 eBioscience
Ly6G FITC 1:500 BD Biosciences
PD-1 BV786 1:200 BD Biosciences

	Tbet BV650 1:200 BD Biosciences<br>EOMES PE 1:200 eBioscience<br>CD80 PE-CF594 1:400 eBioscience<br>CD40 FITC 1:200 BD Biosciences<br>Foxp3 EF450 1:200 eBioscience<br>GATA3 EF660 1:200 eBioscience<br>CXCR3 BV421 1:200 BD Biosciences<br>CXCR6 BV711 1:200 Biolegend<br>KLRG1 PE-DAZZLE 594 1:200 Biolegend<br>CD4 (human) PerCP-Cy5.5 1:50 BD Biosciences<br>CD3 (human) AF700 1:75 BD Biosciences<br>CXCR3 (human) PE-CF594 1:150 BD Biosciences<br>CD45 (human) FITC 1:100 BD Biosciences
Validation	All antibodies were validated by the manufacturers. The P15E tetramer was validated using tumor-infiltrating T cells from mice bearing tumors with and without the P15E antigen.

Eukaryotic cell lines

Policy information about cell lines

Cell line source(s)	CT26 ATCC<br>MC38 generously provided by Dr. Melissa Wong, OHSU<br>4T1 ATCC<br>MCA205-OVA cells generously provided by Dr. Michael Gough, EACRI
Authentication	ATCC validates their cell lines<br>MC38 and MCA205-OVA were validated by the parent labs.
Mycoplasma contamination	All cell lines are tested for Mycoplasma every 15 passages, when there is suspicion of contamination, or when obtained from a new lab/manufacturer.
Commonly misidentified lines<br>(See ICLAC register)	NA

Animals and other organisms

Policy information about studies involving animals; ARRIVE guidelines recommended for reporting animal research

Laboratory animals	C57BL/6, BALB/c, CD8Cre, Lyz2Cre, and Foxp3-eGFP-CreERT2 mice were purchased from the Jackson Laboratories (Bar Harbor, Maine). ALK5flox/flox mice were a generous gift from Andrew Weinberg (Earle A. Chiles Research Institute). All transgenic mice were on C57BL/6 background. Both genders, male and female, were used for experiments with gender matching between control and experimental groups. Mice were between 6-30 weeks of age for experiments.
Wild animals	NA
Field-collected samples	NA
Ethics oversight	The animal facility is OLAW certified (Assurance #D16-00526) and all experiments were carried out under EACRI IACUC protocol #54.

Note that full information on the approval of the study protocol must also be provided in the manuscript.

Human research participants

Policy information about studies involving human research participants

Population characteristics	The TCGA dataset characteristics are publicly available.
Recruitment	*Describe how participants were recruited. Outline any potential self-selection bias or other biases that may be present and how these are likely to impact results.*
Ethics oversight	*Identify the organization(s) that approved the study protocol.*

Note that full information on the approval of the study protocol must also be provided in the manuscript.

Clinical data

Policy information about clinical studies

All manuscripts should comply with the ICMJEguidelines for publication of clinical research and a completedCONSORT checklist must be included with all submissions.

Clinical trial registration	*Provide the trial registration number from ClinicalTrials.gov or an equivalent agency.*
Study protocol	*Note where the full trial protocol can be accessed OR if not available, explain why.*
Data collection	*Describe the settings and locales of data collection, noting the time periods of recruitment and data collection.*
Outcomes	*Describe how you pre-defined primary and secondary outcome measures and how you assessed these measures.*

Flow Cytometry

Plots

Confirm that:

☒ The axis labels state the marker and fluorochrome used (e.g. CD4-FITC).

☒ The axis scales are clearly visible. Include numbers along axes only for bottom left plot of group (a 'group' is an analysis of identical markers).

☒ All plots are contour plots with outliers or pseudocolor plots.

☒ A numerical value for number of cells or percentage (with statistics) is provided.

Methodology

Sample preparation

Spleen, lymph nodes and tumors were harvested from animals and single cell suspensions were prepared using mechanical disaggregation for spleen and lymph nodes only or mincing and enzymatic digestion for 30 min. at 37oC for tumors and lymph nodes for dendritic cell evaluation. Enzyme digest buffer included 1 mg/ml Collagenase A (Roche), 1 mg/ml Hyaluronidase (Sigma) and 50 u/ml DNase (Roche) in DMEM serum free base medium. Following single cell preparation, cells were washed and resuspended in FACS buffer (1x PBS, 1% BSA, 2 mM EDTA) and counted prior to FACS staining using a Guava EasyCyte cytometer (Millipore). Primary T cells were cultured in complete RPMI media (10% heat inactivated FBS, 1% Na-P, 1% NEAA, 10mM HEPES, 55 uM β-mercaptoethanol, and 1% Pen/Strep).

For ex vivo cytokine analysis, cells were first treated with a 1x cell activation cocktail of PMA/ionomycin/Brefeldin A (Biolegend for 5 hours in complete RPMI media. 1x106 cells from single cell suspensions were stained with anti-CD16/CD32 Fc block (1:200, BD Biosciences) and fixable viability 700 dye (1:10,000, BD Biosciences) in 1x PBS for 15 min. at 37C prior to surface and intracellular staining with primary antibodies. Surface staining commenced in 200 ul FACS buffer supplemented with a 1:4 dilution of Brilliant Violet stain buffer (BD Biosciences) and fluorescently conjugated antibodies from the table below for 30 min. at 4C in the dark; all are mouse reactive antibodies unless otherwise indicated. Following surface staining cells were washed and fixed in either 2% PFA or Fix/Perm buffer (eBioscience) for 20 min. at 4oC for intracellular stain. Fix/perm buffer was washed with 1x perm wash buffer (eBioscience) and intracellular proteins were stained with fluorescently conjugated ICS antibodies in perm wash buffer for 30 min. at 4oC in the dark. Cells were washed and resuspended in 1x PBS prior to acquisition on a BD Fortessa or LSRII flow cytometer (BD Biosciences).

Instrument

BD Fortessa or LSRII flow cytometer (BD Biosciences)

Software

FlowJo

Cell population abundance

For CD8 T cell sorting, CD8+ T cells were purified from spleens of naïve CD45.1 C57BL/6 and CD45.2 ALK5aCD8 mice, mixed in equal ratios and labeled with 1 uM CFSE (Molecular Probes) prior to adoptive transfer into C57BL/6 mice bearing MC38 tumors on day 14 post-implant. A portion of the transfered product was used for flow cytometry to confirm purity of sample. Seven days following transfer, tumors, spleens and draining lymph nodes were harvested for FACS analysis.

Gating strategy

The gating strategy for each experiment is listed in the text of the manuscript or figure legend. Where it requires clarification, examples of gating are shown.

☒ Tick this box to confirm that a figure exemplifying the gating strategy is provided in the Supplementary Information.
